# Case Report: Mucus plug in bronchus mimicking a bronchial solid foreign body obstruction

**DOI:** 10.12688/f1000research.12495.1

**Published:** 2017-09-25

**Authors:** Kiran Devkota, Miao He, You Wei Zhang

**Affiliations:** 1 Department of Pediatrics, Renmin Hospital, Hubei University of Medicine, Shiyan, Hubei, 442000, China

**Keywords:** foreign body, bronchial obstruction, plastic bronchitis

## Abstract

Bronchial foreign body obstruction is common in all clinical settings. Obstruction of the airway due to foreign bodies and foreign body aspiration are major causes of childhood mortality and morbidity, which are a big challenge to manage. Occasionally, bronchial obstruction may be due to mucus plugs or other endogenous factors. Here we describe a case of bronchial obstruction caused by mucus plug formation that was managed conservatively in a one-year old boy. The patient was suffering from a cough and noisy breathing for 2 days prior to coming to our hospital, when he experienced sudden onset of difficulty in breathing and a severe cough. At the time of presentation his vital sign readings were:- HR 186 bpm, RR 46/min, BP 78/40 MmHg, temp 36.9°C and SPO2 68%. He was given oxygen immediately and nebulization was started. Chest CT scan was performed that suggested the presence of a right bronchial foreign body with right sided obstructive emphysema. The patient was stable with oxygenation and nebulization with ipratropium bromide, albuterol, normal saline and budesonide before the CT scan. Therefore, we conclude that symptoms resembling foreign body obstruction are not always aspirated or inhaled, and sometimes secreted sputum forms a plug, which mimics the symptoms of foreign body obstruction.

## Introduction

Obstruction of the airway due to foreign bodies, and foreign body aspiration are major causes of childhood mortality and morbidity, which are a big challenge to manage. In the US, more than 17,000 patients visited the emergency department with foreign body aspiration in 2008, and 220 children aged <14 years died due to foreign body aspiration in 2009 ^1^. In children younger than one year, airway obstruction by foreign bodies is the third most common cause of death due to unintentional injury ^1^. In the US, 2900 deaths occur annually due to foreign body aspiration, as estimated by The National Safety Council ^2^. Tracheal and bronchial foreign body obstruction is mainly seen among preschool children:- the most common is in infants and young children. More than 75% of cases due to the foreign body aspiration occur in children aged less than 3 years with 7% mortality rate ^3^. In addition, foreign body aspiration is one of the major causes of infant and childhood mortality in developing countries ^3^. The greatest significance of our case is that it provides another possibility in diagnosis of endogenous foreign bodies and its treatment.

## Case presentation

A one year boy with a history of cough and noisy breathing for 2 days was brought to Renmin Hospital with a sudden onset of difficulty in breathing and severe cough for 5 hours. He had no history of fever, no cold and no history of vomiting or choking. Bowel and bladder habits were normal. He had no significant past medical history. The patient had normal birth history, normal growth, and immunization was up to date. At the time of presentation he was conscious but restless and had difficulty in breathing: more so for inspiration. He had a bluish discoloration of lips, and was pale, but not icteric. He had no signs of dehydration and had no any palpable lymph node. At the time of presentation his vital sign readings were: HR 186 bpm (90–140bpm), RR 46/min (22–37/min), BP 78/40 MmHg (86–106/42–63 MmHg), temp 36.9°C (34.7–37.3°C axillary) and SPO2 68% (95–98%). The throat was hyperemic, grade II enlargement of tonsils with congestion, but no pus point was noted. Chest examination showed bilateral symmetrical chest movement with intercostal retractions. On auscultation there was decreased air entry on the right lung, with wheezing. Heart sounds were not distinct. The abdomen was soft, non-tender. There was no sign of meningitis. Consequently, the patient was initially diagnosed with acute laryngitis with bronchial pneumonia.

As there was history of sudden onset of difficulty in breathing, severe cough, inspiratory dyspnea and decrease air entry on the right lung, we planned for a CT scan of the chest. By this time the patient had been given oxygen, IV fluids, continuous ECG and SPO2 monitoring and nebulization with ipratropium bromide (0.02% /2.5ml), albuterol (0.05mg/kg), normal saline (2ml) and budesonide (0.5mg/2ml). Intravenous methyl prednisolone (2mg/kg/day) was given and antibiotic Meropenem (50mg/kg/day) was started. Routine blood investigations, along with ABG and throat swab cultures were performed. All other investigations were within normal limits, except for ABG: pH 7.25 (7.35–7.45), Pao2 70mmHg (75–100 mmHg), PaCO2 55mmHg (35–45mmHg), HCO3 14.9mmol/L (23–31mmol/L). There was little increase in WBC count 12.93×10 ^9^/L (4–10×10 ^9^/L), Neutrophils 69.4% (50–75%), Lymphocytes 26% (37–52%), Monocytes 2.6%(3–8%) , Eosinophils 1.9%(0.5–5%), Basophils 0.1%(0–1%), and Hb 115gm/L (110–150gm/L).

**Figure 1.**
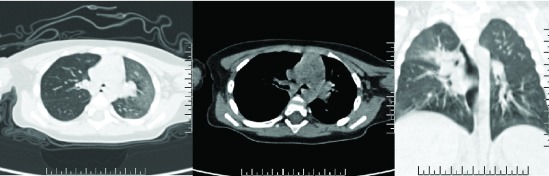
CT scan of the chest showing increased right lung volume and increased transparency of lung field. There is soft tissue density shadow seen in the right main bronchus blocking the lumen. The trachea is shifted slightly to the left, but no obvious abnormity is seen. The chest CT was suggestive of right bronchial foreign body, with right sided obstructive emphysema.

After one and half hours, the patient showed little improvement, and his vital signs were: HR 168 bpm, RR 40/min, BP 76/38 MmHg and SPO2 90% with oxygen via mask. He was continuously on oxygen, IV fluids at maintenance dose, and frequent nebulization with Albuterol (0.05mg/kg TID), ipratropium bromide (0.02% /2.5ml TID), budesonide (0.5mg/2ml BID), and normal saline. Dopamine (5mcg/kg/min) and Dobutamine (2mcg/kg/min) was started on low dose to improve blood circulation. A CT scan of chest was performed that showed increased right lung volume and increased transparency of lung field. A soft tissue density shadow was observed in the right main bronchus blocking the lumen ( Figure 1). The trachea was shifted slightly to the left, but no obvious abnormity was seen. The chest CT was suggestive of the presence of a right bronchial foreign body, with right sided obstructive emphysema.

**Figure 2.**
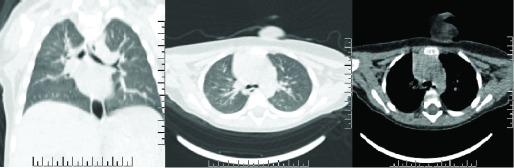
Repeat CT scan of the chest showing infection of the right upper lobe and narrow right upper lobe bronchus.

After the CT scan, a consultation with the Consultant of Otolaryngology and Pulmonologist occurred. The consultant changed the diagnosis to right main bronchus foreign body obstruction probably due to mucus plug, and advised the possible need of a laryngeal mask airway or tracheal intubation, mechanical ventilation and bronchoscopic examination under anesthesia. However, the child was improving gradually, and was much calmer than before, with the exception of noisy breathing with an occasional cough. The patient started feeding after nine hours. The patient’s heart rate decreased to 130bpm RR 36/min and SPO2 92% with oxygen. On auscultation there was b/l conducted sound with wheezing on the right lung. Repeat ABG was pH 7.34, Pao2 85mmHg, PaCO2 46mmHg, HCO3 23.9mmol/L. Methyl prednisolone (2mg/kg/day OD) was continued and the antibiotic was switched to Cefotaxime (100mg/kg/day q12hr). Nebulization was continued and oxygen was given as needed. An additional CT scan of the chest was performed, which showed the infection of the right upper lobe and a narrow right upper lobe bronchus ( Figure 2).

The patient improved gradually and was on same treatment for seven days. Then he was discharged on tapering dose of oral prednisolone. He has not any complained of cough or shortness of breath and chest is clear on auscultation on his follow up after 2 weeks.

## Discussion

Aspiration of a foreign body in the airway mostly occurs in children younger than 15 years; children aged 1–3 years are the most susceptible. Foreign bodies can be exogenous, such as substances inhaled through the mouth (vegetables are the most common airway foreign body); the most common food item that are aspirated are peanuts. In young children, there is lack of molars for proper grinding of food and lack of coordination for swallowing and glottic closure, which is why they are most common age group for foreign body aspiration. Flexible fibro-optic bronchoscopy remains the gold standard for diagnosis and rigid bronchoscopy is the modality of choice in extracting airway foreign bodies ^4^. Endogenous foreign bodies are thick mucus or sputum, bronchial casts, dry scabs, blood clots, pus etc. Endogenous sources may also obstruct airways in same the way as plastic bronchitis ^5^. Plastic bronchitis is a rare disorder, in which there is the presence of gelatinous or rubbery bronchial casts that may be coughed up or found at bronchoscopy or in surgical specimens. Although the pathophysiology is not clearly understood, it is commonly seen in children with congenital heart diseases, cardiac and pericardial diseases in adults and in patients with chronic asthma ^6^. Plastic bronchitis is more common in the lower lobe, but it may also occur in any segments of the bronchial tree ^7^. As plastic bronchitis is common in the lower lobe, it is different to mucoid impaction, which tends to occur in the large segmental bronchi of the upper lobe; they are generally tightly adherent to the wall, and they are retained rather than being expectorated ^7^. Mucoid impaction has a strong correlation with asthma ^8^.

Children cannot cough out the sputum completely. When there is an infection, the glands on the respiratory passage secrete large amounts of mucous and debris, which lead to blockage of respiratory bronchioles and bronchus. This can later develop the symptoms similar to the foreign body obstruction. In the present case, the child presented with symptoms mimicking foreign body obstruction, but the clinical history was not suggestive. Imaging suggested a bronchial foreign body obstruction. By that time the patient’s symptoms were gradually improving by repeated nebulization with normal saline mix with Albuterol and Ipratropium bromide and steroids. This showed us that the sputum may sometimes block the respiratory passage and mimic foreign body bronchial obstruction. In this case we avoided fiberoptic bronchoscopy in order to reduce pain and risk during procedure, as well as reduce medical costs. A limitation in this case was that it is not fully clear whether it was foreign body or blockage due to sputum plug. Post treatment observation is necessary in such a condition. If the block is above the tracheobronchial bifurcation, the child obviously lacks O _2_ and it should be removed immediately by fiberoptic bronchoscope. If the block is below the tracheobronchial bifurcation, the patient can be treated conservatively and observed. Mucolytic agents, like N- Acetyl Cysteine, could have also been used. Salamone *et al*. (2017) published a case report where they mechanically removed bronchial tree-shaped mucous plug in a cystic fibrosis patient ^9^. Park *et al.* mentioned that mucus impaction and plastic bronchitis are usually self-limited or responsive to medical therapy, with a good prognosis, although their patient did not respond to oral corticosteroid or intrabronchial instillation of acetylcysteine and continued to deteriorate despite medical therapy ^8^.

## Conclusion

Symptoms resembling solid foreign body obstruction are not always aspirated or inhaled. Sometimes secreted sputum forming a plug also mimics the symptoms of foreign body obstruction. If there is no clear history of foreign body ingestion or aspiration of gastric content, immediate nebulization may help greatly.

## Consent

We have taken written informed consent from the child’s legal guardian (his Mother) to use her child’s medical case history for the publication of this article.
