# Supplementary material for: Case Report: Mucus plug in bronchus mimicking a bronchial solid foreign body obstruction
Source: F1000Res. 2017 Sep 25;6:1749. [Version 1] doi: 10.12688/f1000research.12495.1 (PMC5871942; doi:10.12688/f1000research.12495.1)
Supplement: Peer review history

# Peer review: Referee response for version 1

**DOI:** 10.5256/f1000research.13529.r31882

**Published:** 2018-03-23

**Authors:** Peng Hu

**Affiliations:** 1 Department of Pediatrics, The First Affiliated Hospital of Anhui Medical University, Hefei, China

Author has made good case report- Mucus plug in bronchus mimicking a bronchial solid foreign body obstruction. We often encounter a child with sudden shortness of breath on emergency department which after nebulization get stable. It would be better if they have done bronchoscopic examination. This case report helps to think other clinician that mucus plug can cause obstruction.

 Author describes the case history, clinical presentation and management in detail. I feel there is space to discuss more on obstruction by other agents.

 I hope this case report is good and help to other clinician as well.

I have read this submission. I believe that I have an appropriate level of expertise to confirm that it is of an acceptable scientific standard.

# Response

**Published:** 2018-03-23

**Authors:** Kiran Devkota

**Affiliations:** Hubei University of Medicine, China

Respected Hu Peng

Thank you so much for your review and valuable suggestion.  

My best regards

Kiran Devkota

# Peer review: Referee response for version 1

**DOI:** 10.5256/f1000research.13529.r32198

**Published:** 2018-03-23

**Authors:** Francesco Menzella

**Affiliations:** 1 Department of Medical Specialties, Pneumology Unit, Arcispedale Santa Maria Nuova - IRCCS, Reggio Emilia, Italy

The case is well described and interesting. I have only a few minor observations:

- On page 3, line 1 and 2, consultant and consultation is repeated several times: I would also use synonyms to avoid repeating the same words
- At least the use of some acronyms must be explicitly expanded, eg ABG, BP, HR
- Line 11 page 3: "b / l" must be written in full

I have read this submission. I believe that I have an appropriate level of expertise to confirm that it is of an acceptable scientific standard.

# Response

**Published:** 2018-03-23

**Authors:** Kiran Devkota

**Affiliations:** Hubei University of Medicine, China

Respected Francesco Menzella

I am very grateful to you. Thank you so much for reveiw and valuable suggestion. 

My best regards

Kiran Devkota
